# The chromosomal genome sequence of the common sea fan, *Gorgonia ventalina* (Linnaeus, 1758) (Malacalcyonacea: Gorgoniidae)

**DOI:** 10.12688/wellcomeopenres.25430.1

**Published:** 2026-01-06

**Authors:** Nikolaos V. Schizas, Jaaziel E. García-Hernández, Jose Victor Lopez, Nina Pruzinsky, Graeme Oatley, Elizabeth Sinclair, Eerik Aunin, Noah Gettle, Camilla Santos, Michael Paulini, Haoyu Niu, Victoria McKenna, Rebecca O’Brien, Wellcome Sanger Institute Tree of Life Management, Samples and Laboratory team, Wellcome Sanger Institute Scientific Operations: Sequencing Operations, Wellcome Sanger Institute Tree of Life Core Informatics team, EBI Aquatic Symbiosis Genomics Data Portal Team, Aquatic Symbiosis Genomics Project Leadership

**Affiliations:** 1 Department of Marine Sciences, University of Puerto Rico, Mayagüez, Puerto Rico; 2 National Coral Reef Institute, Department of Biological Sciences, Nova Southeastern University, Dania Beach, Florida, USA; 3 University Corporation for Atmospheric Research, Boulder, Colorado, USA; 4 NOAA Ocean Exploration Affiliate, Silver Spring, Maryland, USA; 5 Tree of Life, Wellcome Sanger Institute, Hinxton, England, UK

**Keywords:** Gorgonia ventalina; common sea fan; genome sequence; chromosomal; Malacalcyonacea

## Abstract

We present a genome assembly from an individual *Gorgonia ventalina* (common sea fan; Cnidaria; Anthozoa; Malacalcyonacea; Gorgoniidae). The genome sequence has a total length of 339.18 megabases. Most of the assembly (98.66%) is scaffolded into 16 chromosomal pseudomolecules. The mitochondrial genome has also been assembled, with a length of 18.73 kilobases.

## Species taxonomy

Eukaryota; Opisthokonta; Metazoa; Eumetazoa; Cnidaria; Anthozoa; Octocorallia; Malacalcyonacea; Gorgoniidae; *Gorgonia*; *Gorgonia ventalina* (Linnaeus, 1758) (NCBI:txid204384)

## Background

**Figure 1.**
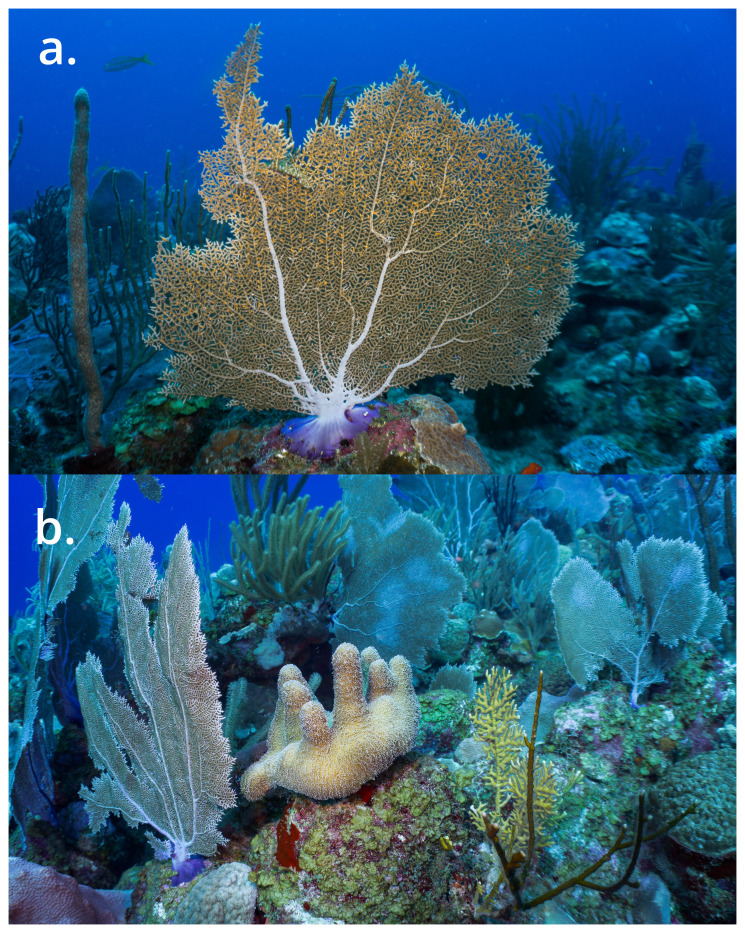
*Gorgonia ventalina* inhabiting shallow and deeper reefs. **a**) The Caribbean purple sea fan. **b**) Scleractinian coral *Dendrogyra cylindrus* surrounded by *G. ventalina*. Photographs by JEGH from La Parguera, Puerto Rico.

One of the most iconic gorgonian species of coral reef ecosystems is the photosynthetic *Gorgonia ventalina* Linnaeus, 1758, commonly known as the purple sea fan ( Figure 1). This soft-coral emerges as one of the most studied and well recognised species inhabiting shallow and deeper coral reefs within the Greater Caribbean and Atlantic region (e.g. Bayer, 1961; Kirk *et al.*, 2009; Rodríguez-Matos *et al.*, 2023). *G. ventalina* belongs to the family Gorgoniidae Lamouroux, 1812, which is comprised of thirteen genera ( Bayer, 1953; McFadden *et al.*, 2025).

Morphologically, *G. ventalina* is characterised by “branches usually compressed in the plane of the fan; ascending branches occasionally somewhat compressed at right angles to the fan, but not the connecting branchlets. Tissue is composed of several spicules; scaphoids with sculpture of convex side reduced to low prickles, sometimes placed on low transverse ridges, spindles acute, anthocodial rods” ( Bayer, 1961: p. 262). *G. ventalina* typically inhabits hydrodynamically dynamic shallow reef ecosystems ( Edmunds, 2020), where it interacts and shares habitat with stony corals, sponges, zoanthids, soft corals, and mobile mesofauna which exploit its morphological structure ( Figure 1) ( McLean & Yoshioka, 2007; Sánchez & Lasker, 2003). Matured coral larvae settle on a viable substrate and grow typically oriented perpendicular to local currents, in an arborescent shape, maximising their nutritional intake, respiration, and photosynthetic needs ( Baker *et al.*, 2015; Rossi *et al.*, 2018).

Generally, diversity, abundance, and densities of Caribbean octocorals tend to decrease with depth (location-dependent), however, their overall ecological importance increases when we consider the recent demise of stony coral populations because of decreased water quality, pathogenic diseases (e.g., stony tissue coral loss disease), coastal development, and high-water temperature related bleaching events ( Goulet & Goulet, 2021; Weil *et al.*, 2009; Williams *et al.*, 2021). Naturally, being sessile, their means of defence against predation and fouling is relied on an array of spicules and chemical compounds (i.e., secondary metabolites), which are produced and coalesced by both the octocoral and its microbiome ( Van Alstyne & Paul, 1992). Despite their chemical protection, several organisms do predate on living tissue of *G. ventalina*, having co-evolved tight host-specific symbiotic relationships with several species of gastropods such as *Coralliophila salebrosa*, *Cymbovula acicularis*, *Cyphoma gibbosum*, *C. signatum*, *Simnialena uniplicata*, and *Tritonia hamnerorum* ( Cronin *et al.*, 1995; Reijnen *et al.*, 2010; Reijnen & van der Meij, 2017; Verboom & Hoeksema, 2023). Polychaetes such as the voracious fireworm, *Hermodice carunculata*, also predate on *G. ventalina* ( Cruz Ramos & Schizas, 2023).

Mortality events have been reported in the past, influenced by high densities (i.e., infestation) of corallivores ( Guzmán & Cortés, 1984; Schärer & Nemeth, 2010) and by highly pathogenic diseases ( Nagelkerken *et al.*, 1997a; Nagelkerken *et al.*, 1997b; Weil *et al.*, 2016), accumulation of detritus/marine snow, and by the overgrowth of other encrusting organisms. Bacterial and fungal pathogens such as *Aspergillus sydowii* (i.e., aspergillosis) have impacted populations of *G. ventalina* across the Caribbean and Atlantic regions ( Bruckner *et al.*, 1997; Dube *et al.*, 2002; Flynn & Weil, 2009). Additionally, recent reports of parasitic epibionts have been discovered affecting *G.ventalina*, including microscopic copepods ( *Sphaerippe* spp.) ( Ivanenko *et al.*, 2017; Korzhavina *et al.*, 2019; Korzhavina *et al.*, 2024; Shelyakin *et al.*, 2018), labyrinthulid protists ( Tracy *et al.*, 2018), including a recently introduced parasitic ophiuroid, *Ophiothela mirabilis* ( Glynn *et al.*, 2020; Hendler *et al.*, 2012), combined, these provide conservation challenges for this foundational species.

Caribbean-wide population differentiation studies have shown that genetic differentiation in the purple sea fan was positively correlated with geographic distance ( Andras *et al.*, 2013). However, some populations of *G. ventalina* were distinct within a few kilometres from each other. As in *G. ventalina* gorgonian host, the genetic patterns of its Symbiodiniaceae were positively correlated with geographic distance, albeit with some different patterns between the two components of the *G. ventalina* holobiont ( Andras *et al.*, 2013). For both the gorgonian host and its symbiotic algae, the Mona Channel was recognised as a significant biogeographic barrier between eastern and western Caribbean populations ( Andras *et al.*, 2011, Andras *et al.*, 2013). This high-quality *G. ventalina* genome will bolster these population level studies, as well as help to address gaps in gorgonian immunology, symbiology, molecular biology and evolution ( Mydlarz *et al.*, 2016; Zhang & Jacobs, 2022).

We present a chromosome-level genome sequence for *Gorgonia ventalina*, the common sea fan. The assembly was produced using the Tree of Life pipeline using a specimen collected from Cayo Enrique, Puerto Rico ( Figure 1). This assembly was generated as part of the Aquatic Symbiosis Genomics project.

## Methods

### Sample acquisition

Sea fan samples were collected by SCUBA within the La Parguera Natural Reserve, Cayo Mario (GPS coordinates: 17.955156, -67.05272) at a depth of 1.5 m on 2021-05-10. Tissue samples were collected under the auspices of the Puerto Rico Department of Natural and Environmental Resources, permit number R-VS-PVS15-SJ-01206-25052021. Small, 1–3 mm subsamples were cut using a sterile scalpel and preserved in RNAlater pre-labeled 2.5 ml cryogenic vials.

The *Gorgonia ventalina* specimen used for genome sequencing had specimen ID NSU0053405 (ToLID jaGorVent5), and a second specimen was used for Hi-C and RNA sequencing (specimen ID NSU0053402, ToLID jaGorVent2).

### Nucleic acid extraction

Protocols for high molecular weight (HMW) DNA extraction developed at the Wellcome Sanger Institute (WSI) Tree of Life Core Laboratory are available on protocols.io ( Howard *et al.*, 2025). The jaGorVent5 sample was weighed and triaged to determine the appropriate extraction protocol. Tissue was homogenised by cryogenic disruption using the Covaris cryoPREP ^®^ Automated Dry Pulverizer. HMW DNA was extracted using the Automated MagAttract v2 protocol. DNA was sheared into an average fragment size of 12–20 kb following the Megaruptor®3 for LI PacBio protocol. Sheared DNA was purified by automated SPRI (solid-phase reversible immobilisation). The concentration of the sheared and purified DNA was assessed using a Nanodrop spectrophotometer and Qubit Fluorometer using the Qubit dsDNA High Sensitivity Assay kit. Fragment size distribution was evaluated by running the sample on the FemtoPulse system.

RNA was extracted from tissue of jaGorVent2 in the Tree of Life Laboratory at the WSI using the RNA Extraction: Automated MagMax™ *mir*Vana protocol. The RNA concentration was assessed using a Nanodrop spectrophotometer and a Qubit Fluorometer using the Qubit RNA Broad-Range Assay kit. Analysis of the integrity of the RNA was done using the Agilent RNA 6000 Pico Kit and Eukaryotic Total RNA assay.

### PacBio HiFi library preparation and sequencing

Library preparation and sequencing were performed at the WSI Scientific Operations core. Libraries were prepared using the SMRTbell Prep Kit 3.0 (Pacific Biosciences, California, USA), following the manufacturer’s instructions. The kit includes reagents for end repair/A-tailing, adapter ligation, post-ligation SMRTbell bead clean-up, and nuclease treatment. Size selection and clean-up were performed using diluted AMPure PB beads (Pacific Biosciences). DNA concentration was quantified using a Qubit Fluorometer v4.0 (ThermoFisher Scientific) and the Qubit 1X dsDNA HS assay kit. Final library fragment size was assessed with the Agilent Femto Pulse Automated Pulsed Field CE Instrument (Agilent Technologies) using the gDNA 55 kb BAC analysis kit.

The sample was sequenced on a Revio instrument (Pacific Biosciences). The prepared library was normalised to 2 nM, and 15 μL was used for making complexes. Primers were annealed and polymerases bound to generate circularised complexes, following the manufacturer’s instructions. Complexes were purified using 1.2X SMRTbell beads, then diluted to the Revio loading concentration (200–300 pM) and spiked with a Revio sequencing internal control. The sample was sequenced on a Revio 25M SMRT cell. The SMRT Link software (Pacific Biosciences), a web-based workflow manager, was used to configure and monitor the run and to carry out primary and secondary data analysis.

### Hi-C

***Sample preparation and crosslinking***

The Hi-C sample was prepared from 20–50 mg of frozen tissue from the jaGorVent2 sample using the Arima-HiC v2 kit (Arima Genomics). Following the manufacturer’s instructions, tissue was fixed and DNA crosslinked using TC buffer to a final formaldehyde concentration of 2%. The tissue was homogenised using the Diagnocine Power Masher-II. Crosslinked DNA was digested with a restriction enzyme master mix, biotinylated, and ligated. Clean-up was performed with SPRISelect beads before library preparation. DNA concentration was measured with the Qubit Fluorometer (Thermo Fisher Scientific) and Qubit HS Assay Kit. The biotinylation percentage was estimated using the Arima-HiC v2 QC beads.

***Hi-C library preparation and sequencing***

Biotinylated DNA constructs were fragmented using a Covaris E220 sonicator and size selected to 400–600 bp using SPRISelect beads. DNA was enriched with Arima-HiC v2 kit Enrichment beads. End repair, A-tailing, and adapter ligation were carried out with the NEBNext Ultra II DNA Library Prep Kit (New England Biolabs), following a modified protocol where library preparation occurs while DNA remains bound to the Enrichment beads. Library amplification was performed using KAPA HiFi HotStart mix and a custom Unique Dual Index (UDI) barcode set (Integrated DNA Technologies). Depending on sample concentration and biotinylation percentage determined at the crosslinking stage, libraries were amplified with 10–16 PCR cycles. Post-PCR clean-up was performed with SPRISelect beads. Libraries were quantified using the AccuClear Ultra High Sensitivity dsDNA Standards Assay Kit (Biotium) and a FLUOstar Omega plate reader (BMG Labtech).

Prior to sequencing, libraries were normalised to 10 ng/μL. Normalised libraries were quantified again to create equimolar and/or weighted 2.8 nM pools. Pool concentrations were checked using the Agilent 4200 TapeStation (Agilent) with High Sensitivity D500 reagents before sequencing. Sequencing was performed using paired-end 150 bp reads on the Illumina NovaSeq 6000.

### RNA library preparation and sequencing

Libraries were prepared using the NEBNext ^®^ Ultra™ II Directional RNA Library Prep Kit for Illumina (New England Biolabs), following the manufacturer’s instructions. Poly(A) mRNA in the total RNA solution was isolated using oligo(dT) beads, converted to cDNA, and uniquely indexed; 14 PCR cycles were performed. Libraries were size-selected to produce fragments between 100–300 bp. Libraries were quantified, normalised, pooled to a final concentration of 2.8 nM, and diluted to 150 pM for loading. Sequencing was carried out on the Illumina NovaSeq X, generating paired-end reads.

### Genome assembly

Prior to assembly of the PacBio HiFi reads, a database of *k*-mer counts ( *k* = 31) was generated from the filtered reads using FastK. GenomeScope2 ( Ranallo-Benavidez *et al.*, 2020) was used to analyse the *k*-mer frequency distributions, providing estimates of genome size, heterozygosity, and repeat content.

The HiFi reads were assembled using Hifiasm ( Cheng *et al.*, 2021) with the --primary option. Haplotypic duplications were identified and removed using purge_dups ( Guan *et al.*, 2020). The Hi-C reads ( Rao *et al.*, 2014) were mapped to the primary contigs using bwa-mem2 ( Vasimuddin *et al.*, 2019), and the contigs were scaffolded in YaHS ( Zhou *et al.*, 2023) with the --break option for handling potential misassemblies. The scaffolded assemblies were evaluated using Gfastats ( Formenti *et al.*, 2022), BUSCO ( Manni *et al.*, 2021) and MERQURY.FK ( Rhie *et al.*, 2020).

The mitochondrial genome was assembled using MitoHiFi ( Uliano-Silva *et al.*, 2023).

### Assembly curation

The assembly was decontaminated using the Assembly Screen for Cobionts and Contaminants ( ASCC) pipeline. TreeVal was used to generate the flat files and maps for use in curation. Manual curation was conducted primarily in PretextView and HiGlass ( Kerpedjiev *et al.*, 2018). Scaffolds were visually inspected and corrected as described by Howe *et al*. (2021). Manual corrections included 94 breaks, 80 joins, and removal of 33 haplotypic duplications. This reduced the scaffold count by 21.8%, increased the scaffold N50 by 27.2%, and reduced the total assembly length by 7.9%. The curation process is described at https://gitlab.com/wtsi-grit/rapid-curation. PretextSnapshot was used to generate a Hi-C contact map of the final assembly.

### Assembly quality assessment

The Merqury.FK tool ( Rhie *et al.*, 2020) was run in a Singularity container ( Kurtzer *et al.*, 2017) to evaluate *k*-mer completeness and assembly quality for the primary and alternate haplotypes using the *k*-mer databases ( *k* = 31) computed prior to genome assembly. The analysis outputs included assembly QV scores and completeness statistics.

The genome was analysed using the BlobToolKit pipeline, a Nextflow implementation of the earlier Snakemake version ( Challis *et al.*, 2020). The pipeline aligns PacBio reads using minimap2 ( Li, 2018) and SAMtools ( Danecek *et al.*, 2021) to generate coverage tracks. It runs BUSCO ( Manni *et al.*, 2021) using lineages identified from the NCBI Taxonomy ( Schoch *et al.*, 2020). For the three domain-level lineages, BUSCO genes are aligned to the UniProt Reference Proteomes database ( Bateman *et al.*, 2023) using DIAMOND blastp ( Buchfink *et al.*, 2021). The genome is divided into chunks based on the density of BUSCO genes from the closest taxonomic lineage, and each chunk is aligned to the UniProt Reference Proteomes database with DIAMOND blastx. Sequences without hits are chunked using seqtk and aligned to the NT database with blastn ( Altschul *et al.*, 1990). The BlobToolKit suite consolidates all outputs into a blobdir for visualisation. The BlobToolKit pipeline was developed using nf-core tooling ( Ewels *et al.*, 2020) and MultiQC ( Ewels *et al.*, 2016), with containerisation through Docker ( Merkel, 2014) and Singularity ( Kurtzer *et al.*, 2017).

### Metagenome assembly

The metagenome assembly was generated using MetaMDBG ( Benoit *et al.*, 2024) and binned using metabat2. PROKKA ( Seemann, 2014) was used to identify tRNAs and rRNAs in each bin, CheckM ( Parks *et al.*, 2015) (checkM_DB release 2015-01-16) was used to assess bin completeness/contamination, and GTDB-Tk ( Chaumeil *et al.*, 2022) (GTDB release 214) was used to taxonomically classify bins.

## Genome sequence report

### Sequence data

**Figure 2.**
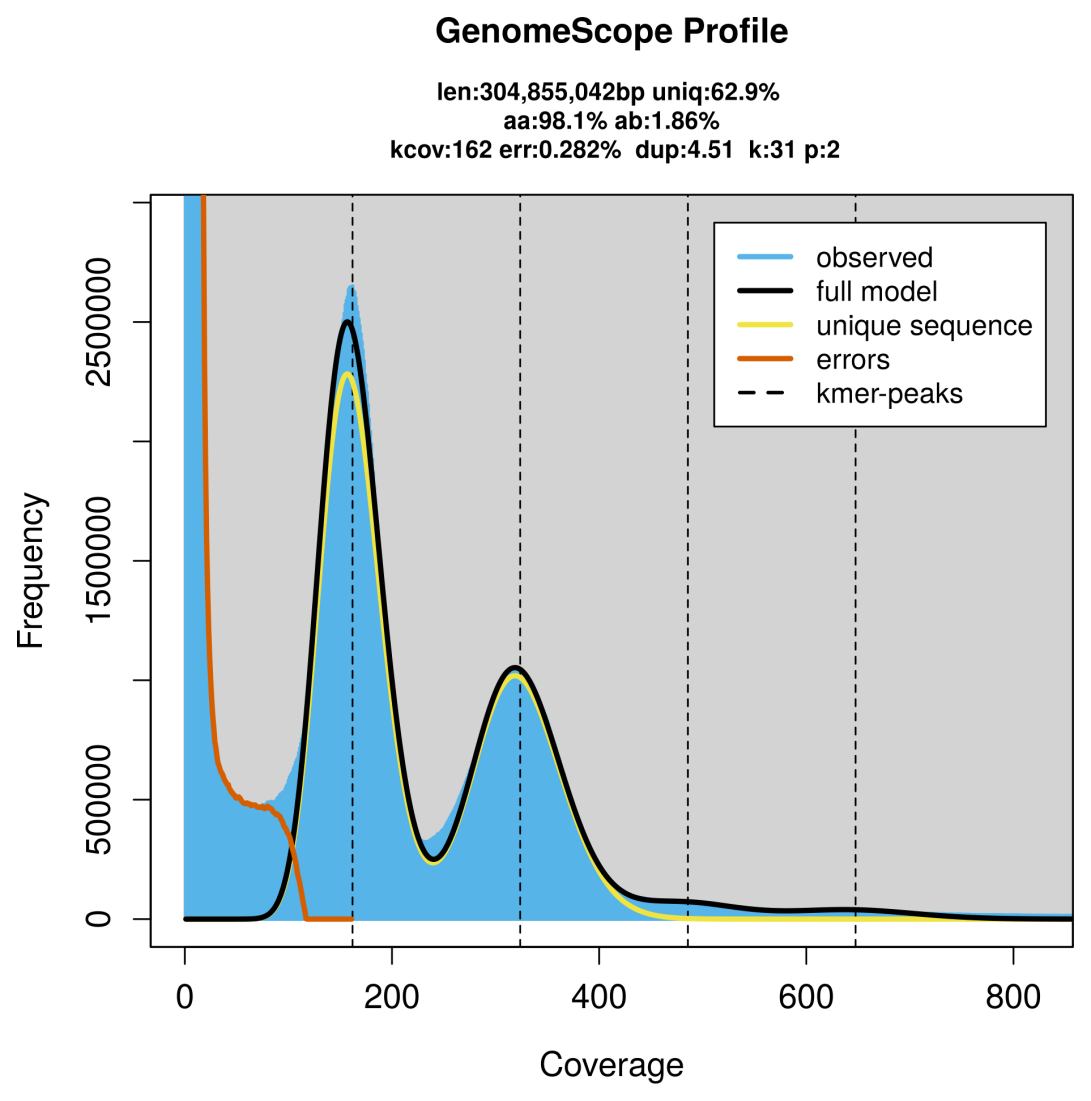
Frequency distribution of *k*-mers generated using GenomeScope2. The plot shows observed and modelled *k*-mer spectra, providing estimates of genome size, heterozygosity, and repeat content based on unassembled sequencing reads.

PacBio sequencing of the *Gorgonia ventalina* specimen generated 115.03 Gb (gigabases) from 18.70 million reads, which were used to assemble the genome. GenomeScope2.0 analysis estimated the haploid genome size at 305.32 Mb, with a heterozygosity of 1.86% and repeat content of 37.14% ( Figure 2). These estimates guided expectations for the assembly. Based on the estimated genome size, the sequencing data provided approximately 59× coverage. Hi-C sequencing produced 331.92 Gb from 2 198.12 million reads, which were used to scaffold the assembly. RNA sequencing data were also generated and are available in public sequence repositories. Table 1 summarises the specimen and sequencing details.

**Table 1.** Specimen and sequencing data for BioProject PRJEB75185.

Platform	PacBio HiFi	Hi-C	RNA-seq
**ToLID**	jaGorVent5	jaGorVent2	jaGorVent2
**Specimen ID**	NSU0053405	NSU0053402	NSU0053402
**BioSample (source individual)**	SAMEA12927233	SAMEA12927230	SAMEA12927230
**BioSample (tissue)**	SAMEA12927281	SAMEA12927278	SAMEA12927278
**Instrument**	Revio	Illumina NovaSeq 6000	Illumina NovaSeq X
**Run accessions**	ERR12954113; ERR13033460; ERR14209105; ERR14209106	ERR12945516; ERR12945514	ERR12945515
**Read count total**	18.70 million	2 198.12 million	42.00 million
**Base count total**	115.03 Gb	331.92 Gb	6.34 Gb

### Assembly statistics

The primary haplotype was assembled, and contigs corresponding to an alternate haplotype were also deposited in INSDC databases. The final assembly has a total length of 339.18 Mb in 128 scaffolds, with 153 gaps, and a scaffold N50 of 19.03 Mb ( Table 2).

**Table 2.** Genome assembly statistics.

**Assembly name**	jaGorVent5.1
**Assembly accession**	GCA_964194065.1
**Alternate haplotype accession**	GCA_964194125.1
**Assembly level**	chromosome
**Span (Mb)**	339.18
**Number of chromosomes**	16
**Number of contigs**	281
**Contig N50**	4.36 Mb
**Number of scaffolds**	128
**Scaffold N50**	19.03 Mb
**Organelles**	Mitochondrion: 18.73 kb

**Figure 3.**
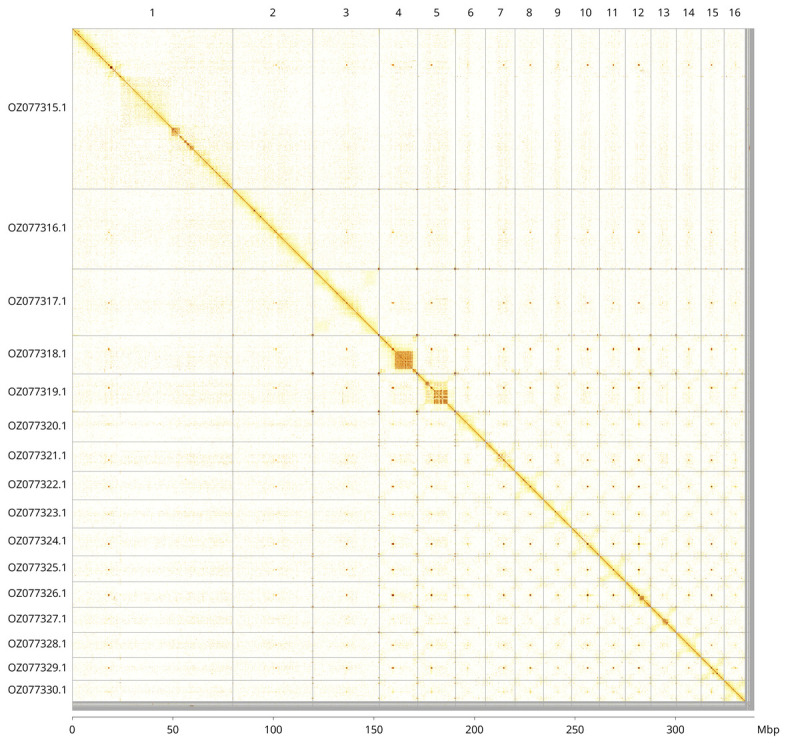
Hi-C contact map of the *Gorgonia ventalina* genome assembly. Assembled chromosomes are shown in order of size and labelled along the axes, with a megabase scale shown below. The plot was generated using PretextSnapshot.

Most of the assembly sequence (98.66%) was assigned to 16 chromosomal-level scaffolds. These chromosome-level scaffolds, confirmed by Hi-C data, are named according to size ( Figure 3; Table 3).

**Table 3.** Chromosomal pseudomolecules in the primary genome assembly of *Gorgonia ventalina* jaGorVent5.

INSDC accession	Molecule	Length (Mb)	GC%
OZ077315.1	1	79.84	36.50
OZ077316.1	2	39.80	36
OZ077317.1	3	33.14	36
OZ077318.1	4	19.03	36.50
OZ077319.1	5	18.85	36.50
OZ077320.1	6	14.96	36
OZ077321.1	7	14.58	36
OZ077322.1	8	14.16	36.50
OZ077323.1	9	13.99	36
OZ077324.1	10	13.84	36
OZ077325.1	11	12.91	36.50
OZ077326.1	12	12.64	36.50
OZ077327.1	13	12.61	36
OZ077328.1	14	12.35	36.50
OZ077329.1	15	11.46	36.50
OZ077330.1	16	10.49	36.50

The mitochondrial genome was also assembled (length 18.73 kb, OZ077331.1). This sequence is included as a contig in the multifasta file of the genome submission and as a standalone record.

### Assembly quality metrics

**Figure 4.**
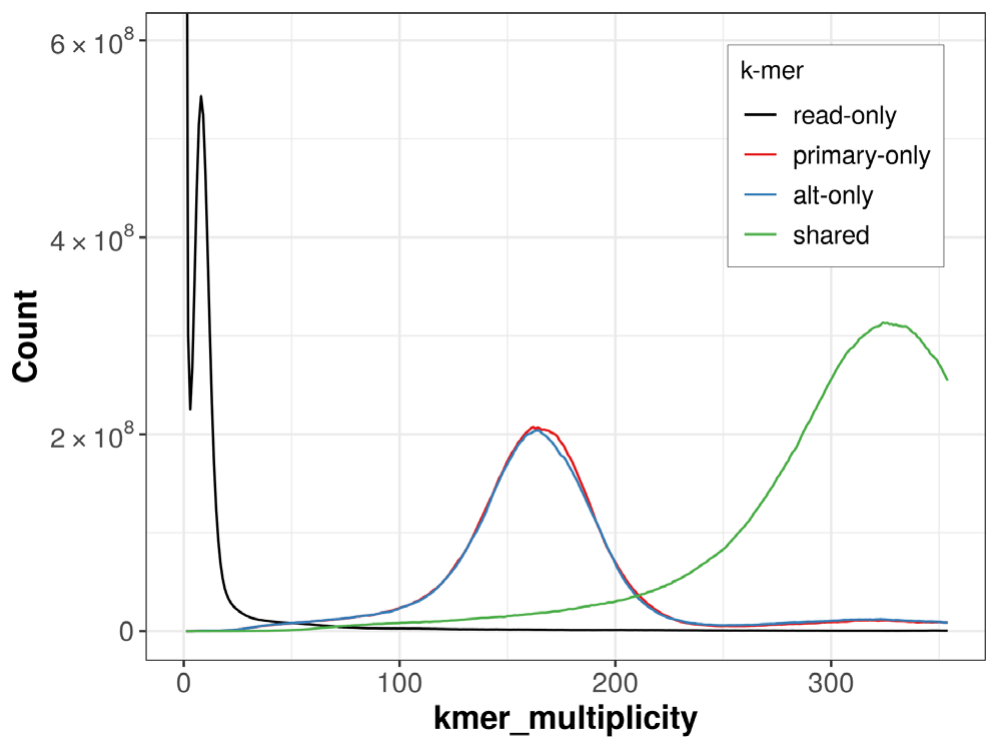
Evaluation of k-mer completeness using MerquryFK. This plot illustrates the recovery of *k*-mers from the original read data in the final assemblies. The horizontal axis represents *k*-mer multiplicity, and the vertical axis shows the number of *k*-mers. The black curve represents *k*-mers that appear in the reads but are not assembled. The green curve corresponds to *k*-mers shared by both haplotypes, and the red and blue curves show *k*-mers found only in one of the haplotypes.

The combined primary and alternate assemblies achieve an estimated QV of 57.8. The *k*-mer completeness is 28.32% for the primary assembly, 28.23% for the alternate haplotype, and 39.69% for the combined assemblies ( Figure 4).

**Figure 5.**
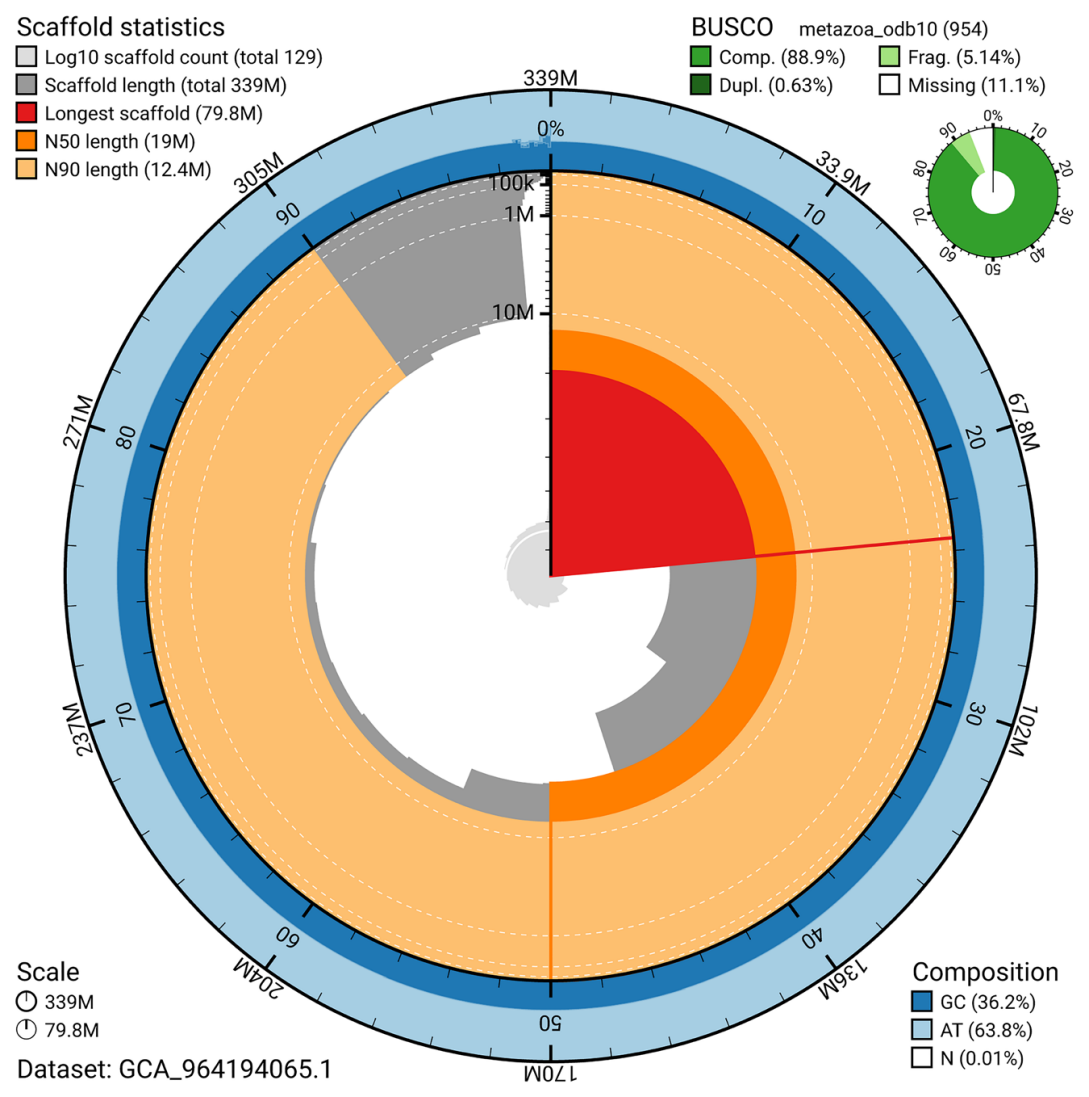
Assembly metrics for jaGorVent5.1. The BlobToolKit snail plot provides an overview of assembly metrics and BUSCO gene completeness. The circumference represents the length of the whole genome sequence, and the main plot is divided into 1 000 bins around the circumference. The outermost blue tracks display the distribution of GC, AT, and N percentages across the bins. Scaffolds are arranged clockwise from longest to shortest and are depicted in dark grey. The longest scaffold is indicated by the red arc, and the deeper orange and pale orange arcs represent the N50 and N90 lengths. A light grey spiral at the centre shows the cumulative scaffold count on a logarithmic scale. A summary of complete, fragmented, duplicated, and missing BUSCO genes in the metazoa_odb10 set is presented at the top right. An interactive version of this figure can be accessed on the BlobToolKit viewer.

**Figure 6.**
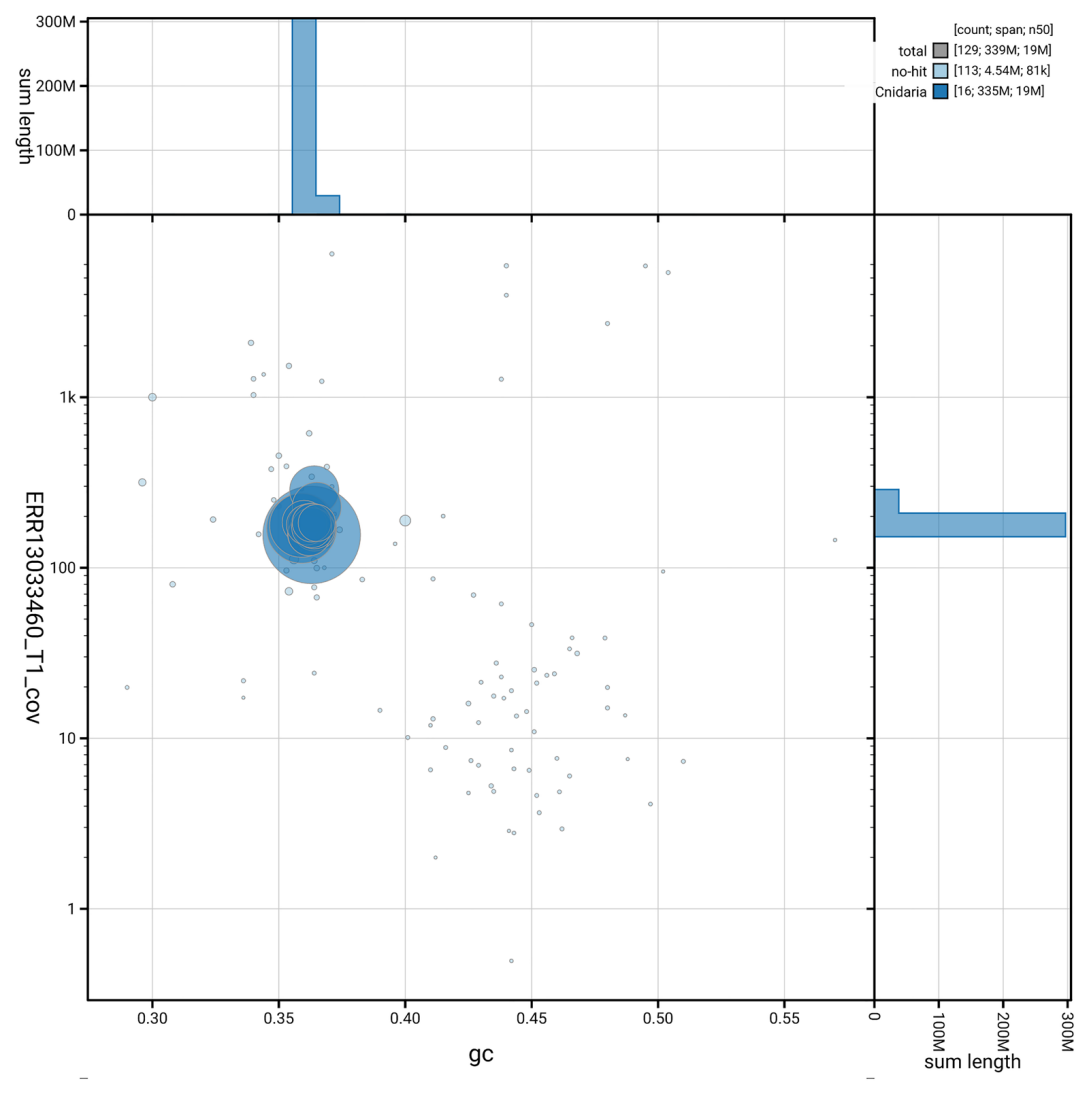
BlobToolKit GC-coverage plot for jaGorVent5.1. Blob plot showing sequence coverage (vertical axis) and GC content (horizontal axis). The circles represent scaffolds, with the size proportional to scaffold length and the colour representing phylum membership. The histograms along the axes display the total length of sequences distributed across different levels of coverage and GC content. An interactive version of this figure is available on the BlobToolKit viewer.

BUSCO v.5.5.0 analysis using the metazoa_odb10 reference set ( *n* = 954) identified 88.9% of the expected gene set (single = 88.3%, duplicated = 0.6%). The snail plot in Figure 5 summarises the scaffold length distribution and other assembly statistics for the primary assembly. The blob plot in Figure 6 shows the distribution of scaffolds by GC proportion and coverage.

Table 4 lists the assembly metric benchmarks adapted from Rhie *et al.* (2021) and the Earth BioGenome Project Report on Assembly Standards September 2024. The EBP metric, calculated for the primary assembly, is **6.C.Q58**, meeting the recommended reference standard.

**Table 4.** Earth Biogenome Project summary metrics for the *Gorgonia ventalina* assembly.

Measure	Value	Benchmark
EBP summary (primary)	6.C.Q58	6.C.Q40
Contig N50 length	4.36 Mb	≥ 1 Mb
Scaffold N50 length	19.03 Mb	= chromosome N50
Consensus quality (QV)	Primary: 58.2; alternate: 57.5; combined: 57.8	≥ 40
*k*-mer completeness	Primary: 28.32%; alternate: 28.23%; combined: 39.69%	≥ 95%
BUSCO	C:88.9% [S:88.3%; D:0.6%]; F:5.1%; M:6.0%; n:954	S > 90%; D < 5%
Percentage of assembly assigned to chromosomes	98.66%	≥ 90%

## Metagenome report

We recovered one bin, assigned to Mycoplasmatales bacterium, from the metagenome assembly, which did not meet the criteria for a MAG. The genome size was 760 kbp, and it was 85.7% complete.

### Wellcome Sanger Institute – Legal and Governance

The materials that have contributed to this genome note have been supplied by a Tree of Life collaborator. The Wellcome Sanger Institute employs a process whereby due diligence is carried out proportionate to the nature of the materials themselves, and the circumstances under which they have been/are to be collected and provided for use. The purpose of this is to address and mitigate any potential legal and/or ethical implications of receipt and use of the materials as part of the research project, and to ensure that in doing so we align with best practice wherever possible.

The overarching areas of consideration are:

- Ethical review of provenance and sourcing of the material
- Legality of collection, transfer and use (national and international)

Each transfer of samples is undertaken according to a Research Collaboration Agreement or Material Transfer Agreement entered into by the Tree of Life collaborator, Genome Research Limited (operating as the Wellcome Sanger Institute) and in some circumstances other Tree of Life collaborators.

## Data Availability

European Nucleotide Archive: Gorgonia ventalina. Accession number PRJEB75185. The genome sequence is released openly for reuse. The *Gorgonia ventalina* genome sequencing initiative is part of the Aquatic Symbiosis Genomics Project (PRJEB43743) and the Sanger Institute Tree of Life Programme (PRJEB43745). All raw sequence data and the assembly have been deposited in INSDC databases. The genome will be annotated using available RNA-Seq data and presented through the Ensembl pipeline at the European Bioinformatics Institute. Raw data and assembly accession identifiers are reported in Table 1 and Table 2. Production code used in genome assembly at the WSI Tree of Life is available at https://github.com/sanger-tol. Table 5 lists software versions used in this study.

## Author information

Contributors are listed at the following links:

- Members of the Wellcome Sanger Institute Tree of Life Management, Samples and Laboratory team
- Members of Wellcome Sanger Institute Scientific Operations – Sequencing Operations
- Members of the Wellcome Sanger Institute Tree of Life Core Informatics team
- Members of the EBI Aquatic Symbiosis Genomics Data Portal Team
- The Aquatic Symbiosis Genomics Project leadership
