# Supplementary material for: The chromosomal genome sequence of the common sea fan, Gorgonia ventalina (Linnaeus, 1758) (Malacalcyonacea: Gorgoniidae)
Source: Wellcome Open Res. 2026 Jan 6;11:14. [Version 1] doi: 10.12688/wellcomeopenres.25430.1 (PMC12856254; doi:10.12688/wellcomeopenres.25430.1)
Supplement: Peer review history

# Reviewer report: Reviewer response for version 1

**DOI:** 10.21956/wellcomeopenres.28016.r144339

**Published:** 2026-02-05

**Authors:** Rodrigo Costa, Matilde Marques

**Affiliations:** 1 Universidade de Lisboa, Lisbon, Lisbon, Portugal; 2 Department of Bioengineering, Universidade de Lisboa Instituto Superior Tecnico, Lisbon, Lisbon, Portugal

Schizas et al. present the chromosomal genome sequence of the sea fan *Gorgonia ventalina *in a coherent and well-structured fashion. The authors provide the rationale underlying the need to sequence the G. ventalina genome, presenting an introduction that covers well the ecology and evolution of this foundational organism. The methodology and genome features are clearly presented and easy to follow. I have a few minor suggestions aiming at improving clarity in a few points.  

 1) For consistency, authors should ideally replace the somewhat informal term “soft coral” with “octocoral” throughout the text to refer to organisms belonging to the Octocorallia class. Authors can also stick to only one, solid term to refer to scleractinian corals throughout.

 2) Correct “stony tissue coral loss disease” with “stony coral tissue loss disease”

 3) modify “predation and fouling is relied on an array of spicules” to “predation and fouling relies on an array…”

 4) The following sentence reads odd as the subject seems to change in the middle of it: “Despite their chemical protection, several organisms do predate on living tissue of G. ventalina, having co-evolved tight host-specific symbiotic relationships with several species of gastropods such as *Coralliophila salebrosa*, *Cymbovula acicularis*…”

 This is not reading well; authors likely want to say that gastropods predate on G. ventalina despite the defence mechanisms of the latter.

 5) The sentence “Bacterial and fungal pathogens such as *Aspergillus sydowii* (i.e., aspergillosis) have impacted populations of *G.* *ventalina*...” can be rephrased for better clarity. *Aspergillus sydowii* (disease-causing agent) and aspergillosis (epizootic condition) are taken as synonyms. Also, aspergillosis can be caused by other Aspergillus species than A. sydowii. A more correct sentence would read “Bacterial and fungal pathogens such as *Aspergillus sydowii* (one of the causative agents of aspergillosis) have impacted populations of *G.* *ventalina…*”

 6) Please revise the following, long sentence for better readability: “Additionally, recent reports of parasitic epibionts have been discovered affecting G. ventalina, including microscopic copepods (Sphaerippe spp.) (Ivanenko et al., 2017; Korzhavina et al., 2019; Korzhavina et al., 2024; Shelyakin et al., 2018), labyrinthulid protists (Tracy et al., 2018), including a recently introduced parasitic ophiuroid, Ophiothela mirabilis (Glynn et al., 2020; Hendler et al., 2012), combined, these provide conservation challenges for this foundational species.”

 Here, “including” is used twice in the same sentence, a space in “G.ventalina” is missing, and a new sentence should start with “combined, …”, as it summarizes the full paragraph.

Are sufficient details of methods and materials provided to allow replication by others?

Yes

Is the rationale for creating the dataset(s) clearly described?

Yes

Are the datasets clearly presented in a useable and accessible format?

Yes

Are the protocols appropriate and is the work technically sound?

Yes

Reviewer Expertise:

Marine symbioses, metagenomics, one health microbiology, microbial ecology, blue biotechnology, bacterial genomics and evolution

We confirm that we have read this submission and believe that we have an appropriate level of expertise to confirm that it is of an acceptable scientific standard.

# Reviewer report: Reviewer response for version 1

**DOI:** 10.21956/wellcomeopenres.28016.r144334

**Published:** 2026-01-29

**Authors:** Jian-Wen Qiu

**Affiliations:** 1 Hong Kong Baptist University, Kowloon, Hong Kong

The authors present a chromosome-level genome assembly for *Gorgonia ventalina*, a common sea fan in the Greater Caribbean and Atlantic, generated as part of the Aquatic Symbiosis Genomics project. They clearly describe the research context and methods, including DNA and RNA extraction; PacBio HiFi library preparation and sequencing; Hi-C sample preparation, library construction, and sequencing; and genome assembly. The final assembly totals 339.18 Mb across 128 scaffolds with 153 gaps and a scaffold N50 of 19.03 Mb. Notably, 98.66% of the sequence is anchored to 16 chromosome-scale scaffolds. Assembly completeness assessed with BUSCO v5.5.0 indicates 88.9% of the expected gene set (single-copy 88.3%, duplicated 0.6%). Metagenome binning recovered one bin assigned to Mycoplasmatales. Overall, the assembly was produced using the Wellcome Sanger Institute's established Tree of Life pipeline; the procedures are well documented, and the results are high quality. I recommend indexing, with two minor suggestions:

 The manuscript notes that the gorgonian is photosynthetic but does not identify the symbiotic algal species. Please add this information.

 Given the photosymbiosis, it is unexpected that metagenome binning recovered only a Mycoplasmatales bacterium and no Symbiodinium. If tissue processing removed symbiotic algae, please state this explicitly in the Methods.

Are sufficient details of methods and materials provided to allow replication by others?

Yes

Is the rationale for creating the dataset(s) clearly described?

Yes

Are the datasets clearly presented in a useable and accessible format?

Yes

Are the protocols appropriate and is the work technically sound?

Yes

Reviewer Expertise:

Comparative genomics, population genetics, phylogenetics of molluscs and annelids.

I confirm that I have read this submission and believe that I have an appropriate level of expertise to confirm that it is of an acceptable scientific standard.

# Reviewer report: Reviewer response for version 1

**DOI:** 10.21956/wellcomeopenres.28016.r144343

**Published:** 2026-01-29

**Authors:** Nabil Amor

**Affiliations:** 1 University Tunis EL Manar, Tunis, Tunisia

Dear Editor,

 This manuscript, titled “ **The chromosomal genome sequence of the common sea fan, *Gorgonia ventalina* (Linnaeus, 1758) (Malacalcyonacea: Gorgoniidae)**”, presents a high-quality chromosome-level genome assembly of *Gorgonia ventalina* generated within the Tree of Life framework.

 The work is technically sound, well documented, and aligns with Earth BioGenome Project standards. The genome will be a valuable resource for octocoral biology, symbiosis research, and conservation genomics. Minor revisions are recommended to improve clarity, consistency, and contextual interpretation of some metrics.

 The background section is strong, but the manuscript would benefit from a short paragraph explicitly linking the genome resource to future applications.

 A brief explanation should be added clarifying why k-mer completeness is low ((28–39%) < the EBP benchmark (≥95%)).

 The metagenome section reports recovery of a single incomplete *Mycoplasmatales* bin. Clarify the rationale for including this section

 Avoid repetition of similar phrasing when describing sequencing platforms.

 Standardise “chromosomal-level scaffolds” vs “chromosome-level scaffolds”.

 When stating that annotation will be available through Ensembl, clarify whether this is planned or already in progress.

 Confirm consistency between Genome size estimate from GenomeScope and final assembly size), with a short explanatory note.

 The manuscript meets the standards of genome resource publication. Addressing the points above will improve interpretability of quality metrics, and biological framing of the dataset.

Are sufficient details of methods and materials provided to allow replication by others?

Yes

Is the rationale for creating the dataset(s) clearly described?

Yes

Are the datasets clearly presented in a useable and accessible format?

Yes

Are the protocols appropriate and is the work technically sound?

Yes

Reviewer Expertise:

Bioinformatics, genomics, phylogenomics

I confirm that I have read this submission and believe that I have an appropriate level of expertise to confirm that it is of an acceptable scientific standard.
